# A rare case of subdural hygroma in an adult male: a clinical image

**DOI:** 10.11604/pamj.2023.44.75.38617

**Published:** 2023-02-07

**Authors:** Taj Afreen Sheikh, Nishigandha Deodhe

**Affiliations:** 1 Neurophysiotherapy Department, Ravi Nair Physiotherapy College, Datta Meghe Institute of Higher Education and Research, Sawangi (Meghe), Wardha, Maharashtra, India

**Keywords:** Physiotherapy, haemorrhage, hygroma, neurological rehabilitation

## Image in medicine

A 59-year-old male was brought to a tertiary care hospital as he met with a road traffic accident 1 month ago, due to which he experienced severe headache, altered speech since a month. The headache was gradual in onset and throbbing in nature. Physical examination was done in which respiratory rate was 18 bpm, pulse rate was 96 bpm, blood pressure 130/90 mmHg. All the investigating procedures were done like complete blood count, computed tomography (CT) for brain. The clinical signs and radiological features, such as subdural hemorrhage (SDH), along with anterior falx cerebri, chronic subdural hygroma, along with convexity of bilateral high parietal and frontal temporal region. With all these findings, the patient was diagnosed with chronic subdural hygroma in bilateral parieto-fronto-temporal region. Subdural hygroma is a condition of accumulation of cerebrospinal fluid in the subdural space following a head trauma. These subdural fluid collections may be clear, pink-tinged, or xanthochromic, may be under variable pressure, and may be found both above or below the tentorium. Signs such as restlessness, stupor, confusion, disorientation, or lethargy can be found. Patient began the treatment with tablets Amlo 5mg once a day, SYP. DUPHALAC 30ml at bedtime, Tab. NATRISE 30mg twice a day, Cap. Felicita OD once a day, Tab. STROCTI P 500mg twice a day, Tab. LEVERA 500mg twice a day, Tab. DOLO 650mg thrice a day, Tab. PAN 40mg once a day, Tab. ZIFI 200mg twice a day. Surgery was suggested for the removal of hygroma. Patient underwent evacuation of hygroma. Patient was managed with antibiotics, analgesics, antacids, anti-epileptic drugs. Post operative check CT brain was done which was satisfactory. Patient was conscious, oriented and vitally, neurologically and haemodynamically stable and shifted to neurosurgery ward. After 1 week mild to moderate level of physiotherapy rehabilitation program was initiated and home exercise program was explained. Patient is being discharged with follow-up plan after 1 month. Following one month of treatment and physiotherapy rehabilitation, the symptoms and quality of life of the patient is improved.

**Figure 1:**
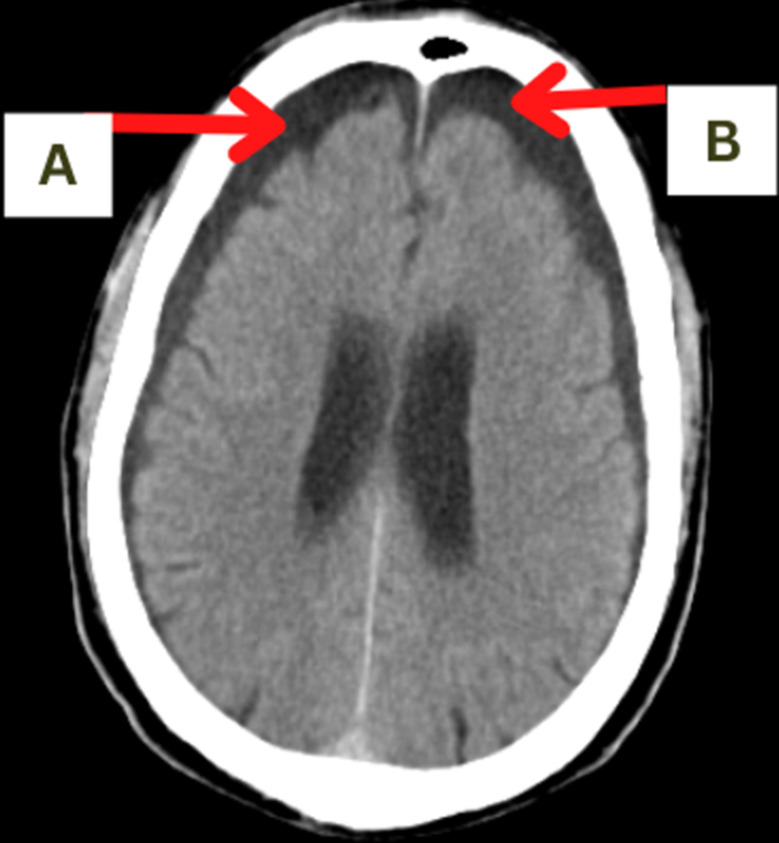
hygroma seen in subdural region indicated by red arrows ‘A’ and ‘B’

